# Dietary Inorganic Nitrate Accelerates Cardiac Parasympathetic Recovery After Exercise in Older Women with Hypertension: A Secondary Analysis of a Randomised Crossover Study

**DOI:** 10.3390/metabo15120789

**Published:** 2025-12-10

**Authors:** Jonas Benjamim, Leonardo Santos Lopes da Silva, Yaritza Brito Alves Sousa, Leonardo da Silva Gonçalves, Guilherme da Silva Rodrigues, Macário Arosti Rebelo, José E. Tanus-Santos, Vitor Engrácia Valenti, Carlos R. Bueno Júnior

**Affiliations:** 1 Institute for Physical Activity and Nutrition (IPAN), Deakin University, Burwood, Melbourne, VIC 3125, Australia; 2 Department of Internal Medicine, Ribeirão Preto Medical School, University of São Paulo, Ribeirão Preto 14049-900, SP, Brazilbuenojr@usp.br (C.R.B.J.); 3 Department of Pharmacology, Faculty of Medical Sciences, State University of Campinas, Campinas 13083-970, SP, Brazil; 4 Department of Pharmacology, Ribeirão Preto Medical School, University of São Paulo (FMRP/USP), Ribeirão Preto 14049-900, SP, Brazil; 5 Autonomic Nervous System Center, Sao Paulo State University, UNESP, Marília 17525-900, SP, Brazil

**Keywords:** nitrate, nitric oxide, exercise, autonomic nervous system, hypertension, cardiovascular physiology

## Abstract

**Background**/**Objectives**: Dietary inorganic nitrate (NO_3_^−^), primarily sourced from vegetables such as beetroot, has been shown to enhance nitric oxide (NO) bioavailability, with emerging evidence suggesting its potential to modulate autonomic function. However, the effects of NO_3_^−^ supplementation on cardiac autonomic recovery post-exercise in hypertensive postmenopausal women remain poorly understood. Using data from a previously conducted randomised controlled trial, this study investigated the effects of acute (800 mg) and seven-day (400 mg/day) beetroot juice NO_3_^−^ supplementation on ultra-short-term post-exercise cardiac parasympathetic recovery in hypertensive older women. **Methods**: In a triple-blind, placebo-controlled crossover design, fourteen postmenopausal women (59 ± 4 y) with hypertension completed two intervention arms (NO_3_^−^ and placebo). Ultra-short-term heart rate variability (HRV) indices (SDNN, RMSSD, HF) were assessed across 5 min post-exercise recovery using 60 s windows. Plasma NO_2_^−^ and NO_3_^−^ concentrations were measured via chemiluminescence. **Results**: Both acute and seven-day NO_3_^−^ supplementation significantly increased plasma NO_2_^−^ and NO_3_^−^ concentrations compared to placebo (*p* < 0.001). Cardiac vagal recovery, assessed via SDNN and RMSSD, was significantly enhanced in both conditions, with greater and more sustained improvements observed after the seven-day protocol. HF power was significantly higher, but only after seven-day supplementation (*p* = 0.009). **Conclusions**: Inorganic NO_3_^−^ supplementation enhances post-exercise cardiac parasympathetic reactivation in hypertensive postmenopausal women. Notably, the seven-day intake (400 mg/day) protocol elicited superior autonomic benefits compared to an acute high dose. These findings highlight the potential of NO_3_^−^ as a non-pharmacological strategy for improving cardiovascular autonomic recovery in high-risk populations.

## 1. Introduction

Inorganic nitrate (NO_3_^−^) obtained from vegetables such as beetroot has been increasingly recognised for its potential role in modulating cardiovascular function through nitric oxide (NO)-dependent pathways [1]. Following ingestion, NO_3_^−^ is initially reduced to nitrite (NO_2_^−^) by commensal bacteria located on the surface of the tongue. The resulting NO_2_^−^ is swallowed and absorbed, while a portion of the remaining NO_3_^−^ is actively transported back to the salivary glands via the sialin transporter and secreted onto the tongue surface, where it can undergo further reduction to NO_2_^−^ [1,2]. Once absorbed in the stomach, NO_2_^−^ can be further reduced to NO in the bloodstream, particularly under hypoxic or acidic conditions [3]. NO generation contributes to systemic physiological effects, including vasodilation and blood pressure regulation [4,5]. Previous studies have suggested NO as a key signalling pathway to induce vascular tone regulation and autonomic nervous system (ANS) balance [6]. Evidence suggests that NO can enhance parasympathetic (vagal) modulation [7], with actions mediated both centrally and peripherally [8]. Studies in animal models have demonstrated that NO donors improve baroreflex sensitivity and vagal modulation [9].

Despite promising results, the interaction between NO_3_^−^ ingestion and ANS remains poorly characterised in populations at elevated cardiovascular risk. Particularly, postmenopausal women with hypertension experience age and hormone-related reductions in vascular compliance and autonomic control, yet are significantly underrepresented in the current literature [10,11]. Most studies focus on acute blood pressure responses in young adults, with few considering autonomic modulation and sex-specific physiological differences [12,13]. Moreover, the comparative influence of acute versus continuous NO_3_^−^ supplementation on cardiac autonomic function remains largely unexplored.

The immediate recovery phase following physical exertion is characterised by rapid shifts in autonomic balance, where timely parasympathetic reactivation and sympathetic withdrawal are crucial for cardiovascular stability [14]. In patients with hypertension or postmenopausal women, the autonomic recovery is often delayed or blunted, reflecting impaired autonomic control and increased susceptibility to adverse cardiovascular events [15,16]. Reduced vagal tone recovery after exercise has been associated with greater morbidity and mortality, independent of resting hemodynamic parameters [17]. Therefore, monitoring autonomic responses to exercise—particularly through tools like heart rate variability (HRV)—provides valuable insights into the functional integrity of the ANS, enabling early detection of dysregulation and supporting risk stratification, therapeutic monitoring, and intervention evaluation in vulnerable populations [18].

In this context, ultra-short-term HRV analysis has emerged as a sensitive and practical tool to assess autonomic recovery, particularly during the immediate post-exercise period. Ultra-short HRV indices may offer increased sensitivity compared to conventional methods, allowing for the capture of rapid fluctuations in autonomic recovery within the initial minutes post-exercise [19]. A previous analysis performed by our group has shown a trend indicating that parasympathetic biomarkers can be used to improve heart rate recovery under increased NO bioavailability. However, it is unclear if this evidence is legitimate due to its lack of statistical significance [20]. Using the same time series analysis, we reproduced the HRV ultra-short-term series to delve into this knowledge. Thus, the integration of ultra-short-term HRV with nutritional interventions such as NO_3_^−^ ingestion may provide novel insights into autonomic regulation and highlight non-pharmacological strategies for cardiovascular risk management in vulnerable populations. This study has been designed to investigate the acute effects and 7-day NO_3_^−^ ingestion on post-exercise cardiac autonomic recovery.

## 2. Materials and Methods

### 2.1. Trial Design

This is a secondary analysis from a triple-blind, placebo-controlled crossover trial [20] that was prospectively registered on ClinicalTrials.gov (NCT05384340) accessed on 1 December 2025 [21] and approved by the Research Ethics Committee of the Ribeirão Preto School of Physical Education and Sport, University of São Paulo, Ribeirão Preto, Brazil (Protocol 55327222.0.0000.5659; 9 March 2022). A sample size analysis has been performed previously for the primary outcome of that study, but not for the variables included in this secondary analysis.

### 2.2. Participants

Postmenopausal women aged 50–65 years (≥12 months of amenorrhea) were recruited via TV, radio, Instagram, and Facebook. Inclusion criteria were as follows: (1) medical diagnosis of hypertension; (2) controlled blood pressure or SBP < 159 mmHg and DBP < 99 mmHg; and (3) clearance for physical activity via the Physical Activity Readiness Questionnaire (PAR-Q). Exclusion criteria included the following: (1) hormone replacement therapy; (2) history of myocardial infarction or stroke; (3) allergy or intolerance to NO_3_^−^; and (4) use of proton pump inhibitors, beta-blockers, or calcium channel blockers.

### 2.3. Before Intervention Assessment

#### 2.3.1. Anthropometry and Body Composition

Participants arrived fasted and in light clothing between 8:00 and 10:00 a.m. Weight and height were measured using a calibrated scale (Welmy^®^ W200, São Paulo, Brazil), and waist/hip circumferences were recorded. Body composition (fat-free mass, fat mass, bone mass) was assessed via Dual X-ray Absorptiometry (Lunar iDXA, GEHealthCare^®^, Chalfont, UK) by a trained technician [22].

#### 2.3.2. Blood Pressure and Cardiopulmonary Exercise Testing (CPET)

Participants completed three laboratory visits on non-consecutive days. In these sessions, blood pressure measurements were performed to assess baseline blood pressure values, which are displayed in Table 1. The blood pressure values were measured indirectly using an ambulatory digital monitor (OMRON-M2^®^, HEM-7121-E, São Paulo, Brazil), previously calibrated on each participant’s left arm. Three measurements were performed at one interval.

Within each visit, three treadmill familiarisation sessions were conducted before undergoing CPET. Participants wore a facial mask on the test day and then performed 30 min of submaximal incremental exercise on a treadmill. The CPET using the modified Bruce protocol [23] consisted of ten 2 min stages with incremental speed or incline. A breath-by-breath gas analyser (Quark PFT, Cosmed^®^, Rome, Italy) was used to determine V̇O_2_peak, defined as the highest 30 s average during the test. All participants achieved a respiratory exchange ratio >1.10 and a heart rate >90% of age-predicted maximum [24]. V̇O_2_peak values were applied to guide the intensity (65–70%) of subsequent submaximal aerobic sessions [21].

### 2.4. Intervention

#### 2.4.1. Inorganic Nitrate Ingestion

Participants were instructed to avoid foods high in NO_3_ established by >15 mg/serving [25], mouthwash (to preserve oral nitrite-producing bacteria) [26], and moderate to vigorous physical activity the day before and on testing days. Dietary intake was standardised via food diaries to be replicated before each evaluation.

The study comprised two seven-day intervention arms: NO_3_^−^ and placebo. On day 1, participants consumed two bottles of beetroot juice (140 mL total) two and a half hours before lab testing; from days 2 to 6, they ingested one bottle with a 70 mL dose daily at home. On day 7, the final dose was taken at home two and a half hours before evaluation. The beetroot juice (Beet-It, James White Drinks Ltd., Ipswich, UK) with NO_3_^−^ contained 6.4 mmol of NO_3_^−^ per 70 mL; the placebo had identical organoleptic characteristics but was NO_3_-depleted (~0.38 mmol) via ion-exchange resin.

Randomisation was performed using a 2:1 ratio on randomizer.org and managed by an independent researcher. Participants crossed over to the alternate intervention after a 7–12-day washout. Juice bottles were consumed while fasting and before toothbrushing. On lab days, participants received a standardised meal, being a whey smoothie (Top Whey 3w, Max Titanium^®^, Brazil; 40 g protein, 4.5 g carbs, 2.0 g fat in 250 mL water) 30 min after beetroot juice ingestion and before testing. Adherence was confirmed via daily photo/video submissions. Blinding was maintained for participants, data collectors, juice distributors, and data analysts, achieving a triple-blind design.

#### 2.4.2. Submaximal Aerobic Exercise

On both the first day and the seventh day of the intervention, participants completed a submaximal aerobic exercise session scheduled two and a half hours after ingesting beetroot juice. Treadmill speed and incline were individually adjusted to elicit and maintain a heart rate corresponding to 65–70% of V̇O_2_peak, continuously monitored via electrocardiogram. This controlled workload served as the standardised physiological stimulus for analysing cardiac autonomic recovery. Each exercise session lasted 30 min.

### 2.5. Outcomes

#### 2.5.1. Cardiac Parasympathetic Recovery Analysis

All assessments began at 8:00 a.m. to control for circadian variation [27]. Environmental temperature and humidity in the laboratory room were kept between 22 and 24 °C and 60–70%, respectively. Participants avoided caffeine (12 h) and alcohol (24 h) before data collection. The HRV technique was employed as an autonomic balance reflection with participants in the supine position, and monitoring of their respiratory rates occurring spontaneously. In this analysis, R-R intervals were continuously monitored on a beat-by-beat basis using a calibrated and validated sensor attached to a heart rate strap with a 1000 Hz sampling frequency (Polar^®^ H10, RS800CX, Helsinki, Finland) [28]. The strap was anatomically positioned just below the externus. R-R intervals were collected during 5 min in a stationary phase. Following each data collection session, the recordings were exported and saved as txt. files. From these recordings, stable segments containing 256 R-R intervals were identified and subjected to both digital and manual artefact removal using PyBios software (v1.0.1, University of São Paulo, Ribeirão Preto, Brazil) [29]. Only data series with over 95% sinus rhythm were retained for analysis [30]. The stationarity of the series was ensured by the manual and visual filtering of ectopic beats and artefacts. The data filtering was performed respecting the correction of up to 5% of the time series. On PyBios software, the default value adopted for the baseline window length was 10, and the tolerance was given a value of 0.1 [29,30].

To compute the HRV metrics, the R-R intervals window was imported into Kubios HRV^®^ software (version 2.1, University of Eastern Finland, Kuopio, Finland) for processing [31]. Time-domain HRV parameters included the root mean square of successive differences (RMSSD) and the standard deviation of normalised R-R intervals (SDNN). Frequency–domain analysis focused on the high-frequency (HF) spectral component (0.15–0.40 Hz), expressed in absolute units (ms^2^). The spectral data were derived using the fast Fourier transform method using 4 Hz [18]. Ultra-short-term analysis was endorsed to precisely analyse parasympathetic recovery modulation in the heart after an exercise session. This validated technique is known as a strong cardiovascular risk predictor as well as a predictor for mortality [28,32]. Each R-R time series previously recorded has been switched into five time-series analyses to perform an ultra-short-term analysis following the window times of 60 s, 120 s, 180 s, 240 s, and 300 s recovery after the exercise. Segmenting the data in this manner enables a high-resolution evaluation of the reactivation of cardiac parasympathetic modulation, which is known to occur rapidly and non-linearly in the early minutes following exercise cessation. Ultra-short-term windows enhance sensitivity to transient fluctuations that may be obscured when using longer averaging intervals and thereby permit a more accurate depiction of HRV recovery kinetics. This approach provides a robust methodological framework for determining the rate and pattern by which autonomic function returns toward baseline after physiological stress.

#### 2.5.2. Blood Collection and Processing

Blood samples were collected via venipuncture at three key time points: before any intervention (baseline), during the rest phase (120 min after beetroot juice (BRJ) ingestion), and at the onset of the recovery phase (immediately post-exercise). For each 5 mL of blood collected in lithium heparin tubes, 500 µL of a stabilisation solution containing 8 mM NEM and 0.1 mM DTPA was added. The samples were promptly centrifuged at 750× *g* for 5 min at 4 °C. Plasma was then separated and rapidly frozen. All plasma samples were stored at –80 °C in amber, UV-resistant tubes until further analysis [33].

#### 2.5.3. NO_3_^−^ and NO_2_^−^ Plasma Analysis

The ozone-based reductive chemiluminescence assay was conducted as previously outlined [34,35]. Plasma aliquots were analysed in duplicate to quantify NO_2_^−^. To determine NO_2_^−^ concentrations, 100 μL of plasma was injected into an acidified tri-iodide solution that was continuously purged with nitrogen and connected to a gas-phase chemiluminescence nitric oxide analyser (Sievers Model 280 NO Analyser, Boulder, CO, USA).

NO_3_^−^ levels were also assessed in duplicate using chemiluminescence following reduction by vanadium(III), as previously reported [20]. In this procedure, plasma samples were introduced into a purge vessel containing vanadium (III) in 1M hydrochloric acid at 90 °C, where NO_3_^−^ was chemically reduced to nitric oxide (NO). The resulting NO gas was transported by nitrogen flow to the NO analyser.

This method relies on the principle of ozone-based reductive chemiluminescence, where NO-related species, such as NO_2_^−^ and NO_3_^−^ compounds that are chemically reduced to NO via tri-iodide or vanadium (III) reagents. Sulfanilamide is added to selectively block interfering species. The liberated NO reacts with ozone (O_3_) to produce a luminescent signal, which is detected and quantified using the eDAQ-Chart software (eDAQ Pty Ltd., v. 5.5.27, Warriewood, NSW 2102, Australia). The intensity of the light emitted corresponds to the concentration of NO released in the gas phase, enabling precise measurement of the targeted analytes [34,35,36].

### 2.6. Statistics

Outliers were initially identified through visual inspection using box plots created in Microsoft Excel^®^. To assess the normality of the data distribution, the Shapiro–Wilk test was applied. All statistical analyses were conducted using RStudio software (version 4.4.0, Posit^®^, Boston, MA, USA), following an intention-to-treat approach. To evaluate the intervention’s effectiveness (NO_3_^−^ vs. placebo), a linear mixed model (LMM) was employed. This model included participants as a random effect, and both time and treatment group as fixed factors, with the treatment serving as the main effect of interest. The intervention type was treated as a between-group factor, while the time points from the recovery phases were treated as repeated measures. Bonferroni correction was applied for multiple comparisons in the recovery phase within each time point. The LMM was used to assess treatment effects on autonomic recovery responses at rest. Intergroup comparisons (placebo vs. NO_3_^−^) for plasma NO_2_^−^ and NO_3_^−^ concentrations were analysed using either an independent *t*-test or the Mann–Whitney U test, depending on data distribution. A significance level of *p* < 0.05 was adopted for all analyses. HRV, NO_2_^−^, and NO_3_^−^ values are presented as means and standard error of the mean (SEM).

## 3. Results

### 3.1. Participants

**Figure 1:**
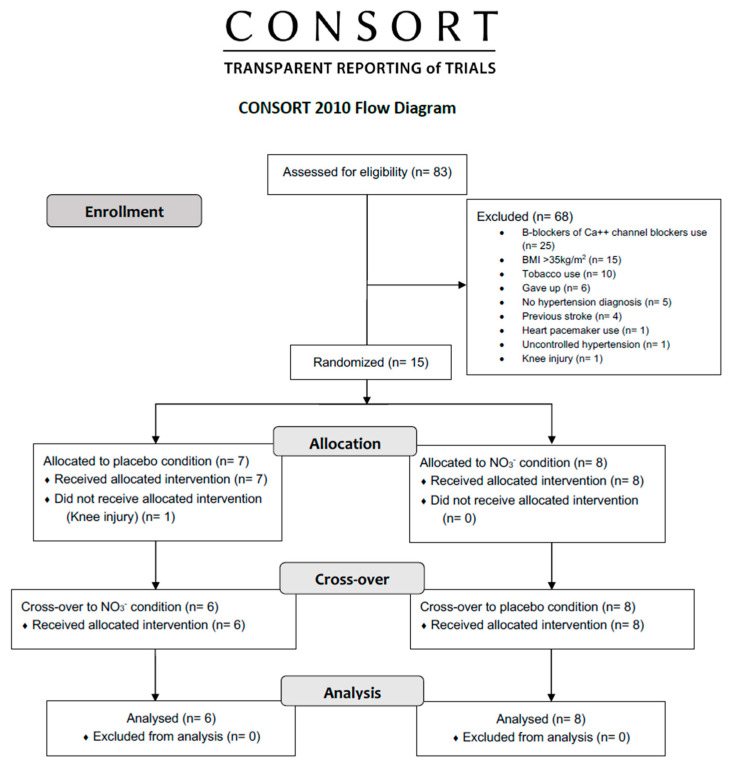
Flowchart demonstrating screening, allocation, and analysis of trial phases.

Fourteen postmenopausal women [mean (SEM) age: 59 (1) y; BMI (kg/m^2^): 29 (0.8); V̇O_2_peak (mL.kg^−1^.min^−1^): 24.1 (0.58)] with previous systemic arterial hypertension (SAH) diagnoses (15 (3.2) y) completed the study protocols (Figure 1). Further participants’ health baseline data are described in Table 1. Eleven participants reported experiencing beeturia (reddish discoloration of urine or stool), a known benign effect of beetroot ingestion. Two participants reported mild discomfort related to the sweet taste of the juice during initial consumption, which resolved spontaneously after the first few servings.

The most prevalent pharmacological therapy to treat SAH in this population was diuretics (70%), followed by angiotensin-converting enzyme (ACE) inhibitors (50%) and angiotensin-1 receptor blockers (21%). Five participants underwent therapy to control cholesterol; three participants had been using anti-diabetic drugs, and two used anti-depressants.

### 3.2. Nitric Oxide Stable Metabolites Plasma Concentrations

Two and a half hours after the ingestion of beetroot juice rich in NO_3_^−^, measurements of plasma NO_2_^−^ concentrations (0.07 μM (0.01) revealed an increase of ≈13-fold after acute intervention and ≈7-fold after seven-day intervention in comparison to baseline values. NO_3_^−^ plasma values (19.8 μM (1.5) had also increased by ≈23-fold after acute intervention and by ≈19-fold following seven-day intervention with beetroot juice rich in NO_3_^−^. The differences in NO_2_^−^ and NO_3_^−^ plasma concentration measurements between the placebo vs. NO_3_^−^ protocols can be observed in detail in Table 2.

**Figure 2:**
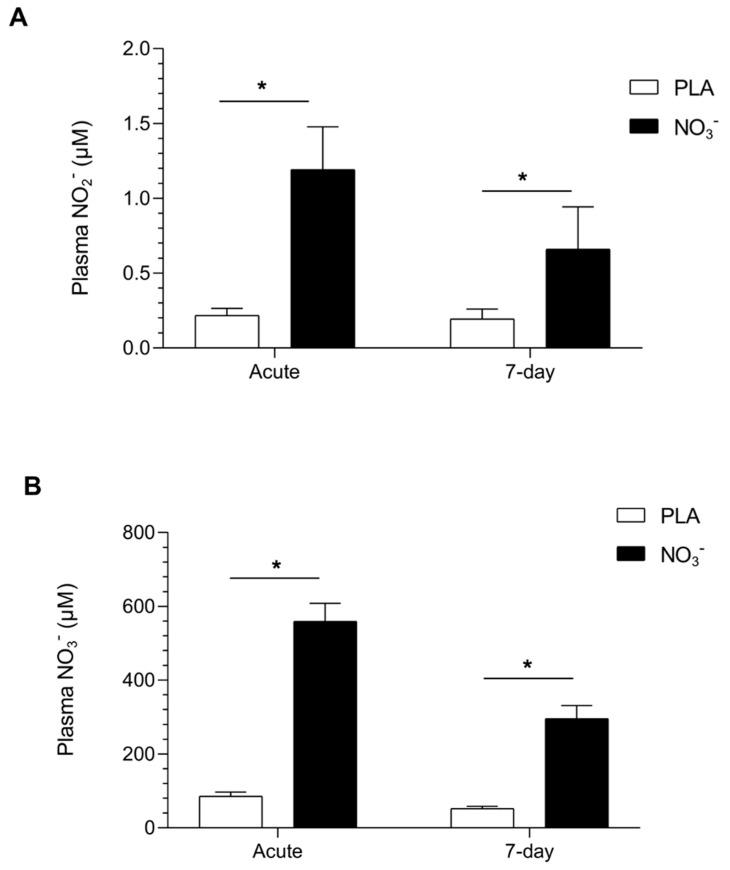
Effects of beetroot juice (NO_3_^−^) ingestion on NO_2_^−^ and NO_3_^−^ plasma concentrations after submaximal aerobic exercise. (**A**) Plasma nitrite (NO_2_^−^) values; (**B**) Plasma nitrate (NO_3_^−^) values; Acute: 800 mg; 7-day: 400 mg; PLA: Placebo; NO_3_^−^: Beetroot juice rich in NO_3_^−^. Data is presented with mean and SEM. Differences between group conditions are compared (placebo vs. NO_3_^−^) at the same time point; *t*-test or Mann–Whitney non-parametric approach used as appropriate (* *p* < 0.001).

After submaximal aerobic exercise, measurements of NO_2_^−^ and NO_3_^−^ plasma concentrations in the placebo vs. NO_3_^−^ group were significantly different. NO_2_^−^ plasma revealed significant changes after ingestion of an acute dose of beetroot juice rich in NO_3_^−^ [1.19 µM (0.26), *p* < 0.001] in comparison to the placebo group [0.21 µM (0.04)]. NO_3_^−^ also showed a significant increase after ingestion of an acute dose of beetroot juice rich in NO_3_^−^ [559.2 µM (47.25), *p* < 0.001] in comparison to the placebo group [85.7 µM (10.34)]. After seven days, the plasma NO_2_^−^ values [0.66 (0.25), *p* < 0.001] of participants who had consumed beetroot juice rich in NO_3_^−^ were higher than those of the placebo group [0.19 (0.23), *p* < 0.001]. In the same manner, NO_3_^−^ plasma values [296.1 (27.87), *p* < 0.001] were also higher than in the placebo group [52.3 (5.87), *p* < 0.001] (Figure 2).

### 3.3. Cardiac Parasympathetic Recovery After Exercise

**Figure 3:**
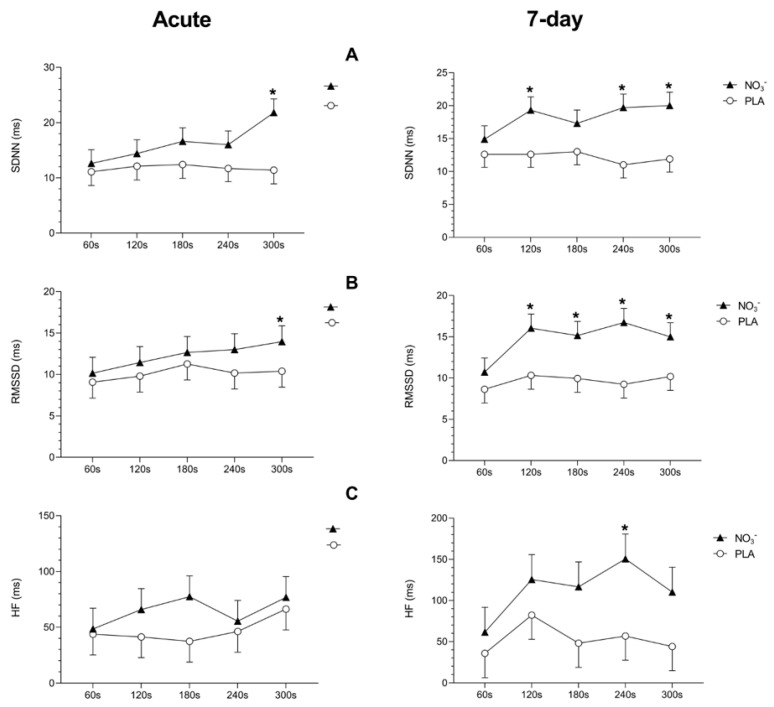
Effects of beetroot juice rich in NO_3_^−^ on ultra-short-term HRV parasympathetic indexes following submaximal aerobic exercise. Data presented with mean and SEM values. * Between-group significant differences using linear mixed models (treatment effect, *p* < 0.05); (**A**) SDNN: standard deviation of R-R normalised (NN) intervals. (**B**) RMSSD: root-mean-square of R-R intervals successive differences; (**C**) HF: high-frequency spectral index (HF: 0.15–0.40 Hz) in absolute units (ms).

Following acute ingestion of NO_3_^−^-rich beetroot juice (800 mg NO_3_^−^), indices of vagal modulation showed significant improvements compared to the placebo condition. The SDNN (ms) index was significantly higher at 300 s of recovery in the NO_3_^−^ condition [21.8 (2.49)] versus the placebo [11.4 (2.49); *p* < 0.001]. Similarly, RMSSD (ms) values were elevated at 300 s following NO_3_^−^ ingestion [13.96 (1.92)] compared to the placebo [10.39 (1.92); *p* = 0.04]. No significant between-group differences were observed for the HF (ms^2^) index in the acute condition. These findings are illustrated on the left side of Figure 3 (Acute and 7-Day Protocols), with complete *p*-values and 95% confidence intervals provided in the Supplementary Material.

After seven days of continuous beetroot juice ingestion (400 mg NO_3_^−^/day), cardiac parasympathetic recovery was again enhanced in the NO_3_^−^ condition compared to the placebo. Notably, the magnitude and consistency of the improvements were greater than those observed after acute ingestion. Between-group comparisons revealed significantly higher SDNN (ms) values in the NO_3_^−^ vs. placebo group at 120 s [19.3 (2.04) vs. 12.6 (2.00); *p* = 0.003], 240 s [19.7 (2.04) vs. 11.0 (1.99); *p* < 0.001], and 300 s [20.0 (2.04) vs. 11.9 (2.00); *p* < 0.001] of recovery. RMSSD (ms) also demonstrated significant group differences, with higher values in the NO_3_^−^ condition at 120 s [16.0 (1.71) vs. 10.3 (1.67); *p* = 0.0012], 180 s [15.1 (1.71) vs. 9.94 (1.70); *p* = 0.003], 240 s [16.7 (1.70) vs. 9.24 (1.67); *p* < 0.001], and 300 s [15.0 (1.71) vs. 10.2 (1.67); *p* = 0.005]. Furthermore, the HF (ms^2^) index also supported improved parasympathetic modulation following seven-day NO_3_^−^ supplementation, with significantly higher values at 240 s in the NO_3_^−^ group [150.5 (30.3)] compared to placebo [56.7 (29.4); *p* = 0.009]. These findings are illustrated on the right side of Figure 3 (Acute and 7-Day Protocols), with complete *p*-values and 95% confidence intervals provided in the Supplementary Material.

## 4. Discussion

This study evaluated the effects of both acute and seven-day NO_3_^−^ supplementation on autonomic nervous system modulation in postmenopausal women diagnosed with hypertension, using aerobic exercise as a physiological stressor. Our key finding is that, while a single acute dose of 800 mg NO_3_^−^ improved cardiac autonomic recovery after exercise, a lower daily dose of 400 mg administered over seven days produced even greater benefits, particularly in the reestablishment of vagal modulation. These results suggest that repeated dosing may improve the autonomic response to exercise more effectively than a single higher dose, despite the lower cumulative NO_3_^−^ intake.

Both protocols significantly elevated plasma concentrations of NO_2_^−^ and NO_3_^−^, confirming absorption and a systemic increase in stable NO products. Notably, the seven-day protocol elicited more consistent and sustained improvements in HRV indices, including SDNN, RMSSD, and HF power, during the recovery phase. These indices reflect parasympathetic modulation and are crucial for cardiovascular recovery following physical exertion, especially in populations with autonomic dysfunction.

The superior autonomic outcomes observed with the 7-day protocol may be attributed to cumulative physiological adaptations associated with sustained NO exposure. While acute NO_3_^−^ intake can rapidly increase NO availability, continuous moderate-dose supplementation may promote longer-lasting endothelial and neural adjustments, including improved baroreflex sensitivity, reduced sympathetic tone, and enhanced parasympathetic responsiveness. These mechanisms are particularly relevant in postmenopausal women, where oestrogen deficiency impairs endothelial NO synthase (eNOS) activity, contributing to arterial stiffness and blunted vagal activity [37].

Our findings align with and extend prior studies showing that NO_3_^−^ supplementation reduces blood pressure and improves vascular function in patients with hypertension [4,38,39,40]. However, few studies have explored NO_3_^−^’s role in modulating the autonomic nervous system, and even fewer have assessed these effects in postmenopausal women with hypertension. Notay et al. [41] previously reported reductions in muscle sympathetic nerve activity following NO_3_^−^ ingestion, supporting the hypothesis that NO_3_^−^ may influence central autonomic regulation. Our data provide novel evidence that such effects are not limited to resting conditions but extend to dynamic autonomic recovery following exercise.

The clinical implications of these findings are substantial. Impaired autonomic recovery after physical exertion is a marker of poor cardiovascular prognosis and increased mortality in individuals with hypertension [17]. By demonstrating that seven-day NO_3_^−^ supplementation can accelerate parasympathetic reactivation—despite a lower daily dose—our study suggests a practical and accessible non-pharmacological strategy to improve autonomic function and cardiovascular adaptability in high-risk populations. The present findings also carry potential clinical relevance when considering the prognostic importance of autonomic recovery after exercise. Although the absolute changes in RMSSD and SDNN during the post-exercise period were relatively small, they nonetheless reflect consistent improvements in cardiac vagal reactivation across consecutive recovery epochs. This pattern is noteworthy given that post-exercise autonomic recovery—particularly vagal re-engagement—is a recognised predictor of cardiovascular morbidity and mortality, independent of resting blood pressure or baseline HRV. Hypertensive postmenopausal women typically present with attenuated parasympathetic responsiveness; thus, even modest enhancements in HRV-derived indices during early recovery may represent physiologically meaningful shifts in autonomic balance. Accordingly, our results suggest that short-term dietary NO_3_^−^ supplementation has the potential to positively influence a clinically relevant marker of cardiovascular prognosis, supporting further investigation in larger cohorts and longer-term interventions.

The strengths of this study include its randomised, triple-blind design, the use of validated HRV measures with ultra-short-term analysis for fine-resolution tracking of post-exercise autonomic shifts, and strict control over confounding variables such as diet, physical activity, and oral NO_3_^−^ metabolism. Noteworthily, we monitored adherence and ensured participants avoided behaviours and substances (e.g., mouthwash, proton pump inhibitors) known to interfere with NO_3_^−^-NO2^−^-NO pathway metabolism.

A key methodological strength of this study was the use of ultra-short-term HRV analysis, which may explain, at least in part, our ability to detect significant differences between groups. While previous studies [20,42] failed to identify meaningful changes in autonomic modulation following NO_3_^−^ supplementation in similar populations, our approach captured rapid and subtle fluctuations in parasympathetic activity during the initial minutes of post-exercise recovery—a physiologically dynamic and clinically relevant window. This high-resolution temporal analysis offered a more nuanced understanding of the autonomic effects of NO_3_^−^, particularly for postmenopausal women with hypertension [20]. Therefore, our findings suggest that ultra-short-term HRV assessments represent a more sensitive and comprehensive tool for evaluating interventions aimed at restoring autonomic balance in high cardiovascular risk populations.

This study presents some limitations that warrant consideration. First, the relatively small sample size, although statistically powered based on pilot data, limits the generalizability of our findings. While the crossover design helped to mitigate inter-individual variability, the modest number of participants may not capture the full spectrum of physiological responses. Our sample consisted of postmenopausal women with controlled hypertension, excluding individuals with more complex comorbidities or those on varied pharmacological regimens. As such, the findings may not be directly applicable to broader clinical populations, particularly those undergoing polypharmacy or experiencing severe cardiovascular dysfunction. Additionally, our sample excluded individuals taking β-blockers or calcium channel blockers to avoid interference with cardiac autonomic measurements. While this choice increased the internal validity of our autonomic measures, it may limit generalizability to the broader population with hypertension. Lastly, the effectiveness of NO_3_^−^ supplementation over periods longer than seven days remains to be established and should be evaluated in future trials.

## 5. Conclusions

Both acute and short-term supplementation with BRJ-NO_3_^−^ improved cardiac autonomic recovery following exercise in postmenopausal women with SAH. Notably, the seven-day low-dose protocol elicited superior parasympathetic benefits compared to a single high-dose intervention, highlighting the potential for sustained NO_3_^−^ intake to support cardiovascular autonomic health in this vulnerable population.

## Figures and Tables

**Table 1:** Baseline participant health variables are presented with mean and SEM values.

Variables	Values
Height (m)	1.56 (0.02)
Mass (kg)	71.2 (2.5)
Fat-free mass (kg)	39.8 (1.3)
Fat mass (kg)	30.7 (1.6)
Visceral fat (kg)	2.59 (0.2)
BMQE (score)	4.54 (0.4)
Cholesterol (mmol/L)	3.87 (0.2)
HDL (mmol/L)	0.87 (0.05)
LDL (mmol/L)	2.5 (0.2)
TGL (mmol/L)	2.6 (0.4)
Plasma NO_3_^−^ (μM)	19.8 (1.5)
Plasma NO_2_^−^ (μM)	0.07 (0.01)
SBP/DBP (mmHg)	137 (4)/82 (2.4) (Visit 1)
	137 (3.4)/83 (1.8) Visit 2)
	138 (3.7)/82 (2.1) (Visit 3)

Legend: mmol/L: millimoles per litre; μM: micromole; kg: kilogram; m: metres; mmHg: millimetre of mercury; HDL: high-density lipoprotein; LDL: low-density lipoprotein; TGL: triglycerides; SBP: systolic blood pressure; DBP: diastolic blood pressure; BMQE: Baecke modified questionnaire for older people.

**Table 2:** NO_2_^−^ and NO_3_^−^ plasma 120 min after beetroot juice ingestion with or without NO_3_^−^ following acute and 7-day intervention. Data presented with mean and SEM values.

Variables	Acute			7-Day		
	Placebo	NO_3_^−^	*p*-Value	Placebo	NO_3_^−^	*p*-Value
NO_2_^−^ (μM) Plasma	0.19 (0.03)	0.93 (0.18) *	<0.001	0.19 (0.05)	0.48 (0.1) *	<0.001
NO_3_^−^ (μM) Plasma	71.7 (7.4)	465 (58.5) *	<0.001	74.3 (11)	382 (26.4) *	<0.001

* Differences between intergroup conditions (placebo vs. nitrate), *t*-test, or Mann–Whitney non-parametric approach as appropriate, *p* < 0.05.

## Data Availability

Further inquiries can be directed to the corresponding author. Data raw will be shared when requested.

## Supplementary Materials

The following supporting information can be downloaded at: https://www.mdpi.com/article/10.3390/metabo15120789/s1.
