# Supplementary material for: Dietary Inorganic Nitrate Accelerates Cardiac Parasympathetic Recovery After Exercise in Older Women with Hypertension: A Secondary Analysis of a Randomised Crossover Study
Source: Metabolites. 2025 Dec 10;15(12):789. doi: 10.3390/metabo15120789 (PMC12734956; doi:10.3390/metabo15120789)
Supplement: Supplementary file 1 [file metabolites-15-00789-s001.zip › metabolites-4008106-supplementary.pdf]

SDNN (Acute)

> emm_sdnna

Group	Time	emmean	SE	df	lower.CL	upper.CL
NO3	T1	12.6	2.49	59.5	7.62	17.6
PLA	T1	11.1	2.49	59.5	6.12	16.1
NO3	T2	14.4	2.49	59.5	9.46	19.4
PLA	T2	12.1	2.49	59.5	7.11	17.1
NO3	T3	16.6	2.49	59.5	11.58	21.6
PLA	T3	12.4	2.49	59.5	7.46	17.4
NO3	T4	16.0	2.49	59.5	11.03	21.0
PLA	T4	11.7	2.49	59.5	6.70	16.7
NO3	T5	21.8	2.49	59.5	16.77	26.7
PLA	T5	11.4	2.49	59.5	6.43	16.4

Linear mixed model

> pairs(emm_sdnna, adjust = "none")

contrast	estimate	SE	df	t.ratio	p.value
NO3 T1 - PLA T1	1.500	2.81	117	0.533	0.5949
NO3 T1 - NO3 T2	-1.836	2.81	117	-0.653	0.5153
NO3 T1 - PLA T2	0.507	2.81	117	0.180	0.8573
NO3 T1 - NO3 T3	-3.964	2.81	117	-1.409	0.1614
NO3 T1 - PLA T3	0.164	2.81	117	0.058	0.9535
NO3 T1 - NO3 T4	-3.407	2.81	117	-1.211	0.2283
NO3 T1 - PLA T4	0.921	2.81	117	0.328	0.7439
NO3 T1 - NO3 T5	-9.150	2.81	117	-3.252	0.0015
NO3 T1 - PLA T5	1.186	2.81	117	0.421	0.6742
PLA T1 - NO3 T2	-3.336	2.81	117	-1.186	0.2381
PLA T1 - PLA T2	-0.993	2.81	117	-0.353	0.7248
PLA T1 - NO3 T3	-5.464	2.81	117	-1.942	0.0545
PLA T1 - PLA T3	-1.336	2.81	117	-0.475	0.6358
PLA T1 - NO3 T4	-4.907	2.81	117	-1.744	0.0837
PLA T1 - PLA T4	-0.579	2.81	117	-0.206	0.8374
PLA T1 - NO3 T5	-10.650	2.81	117	-3.786	0.0002
PLA T1 - PLA T5	-0.314	2.81	117	-0.112	0.9112
NO3 T2 - PLA T2	2.343	2.81	117	0.833	0.4067
NO3 T2 - NO3 T3	-2.129	2.81	117	-0.757	0.4508
NO3 T2 - PLA T3	2.000	2.81	117	0.711	0.4786
NO3 T2 - NO3 T4	-1.571	2.81	117	-0.559	0.5775
NO3 T2 - PLA T4	2.757	2.81	117	0.980	0.3291
NO3 T2 - NO3 T5	-7.314	2.81	117	-2.600	0.0105
NO3 T2 - PLA T5	3.021	2.81	117	1.074	0.2850
PLA T2 - NO3 T3	-4.471	2.81	117	-1.589	0.1147
PLA T2 - PLA T3	-0.343	2.81	117	-0.122	0.9032
PLA T2 - NO3 T4	-3.914	2.81	117	-1.391	0.1668
PLA T2 - PLA T4	0.414	2.81	117	0.147	0.8832
PLA T2 - NO3 T5	-9.657	2.81	117	-3.433	0.0008
PLA T2 - PLA T5	0.679	2.81	117	0.241	0.8098
NO3 T3 - PLA T3	4.129	2.81	117	1.468	0.1449
NO3 T3 - NO3 T4	0.557	2.81	117	0.198	0.8434
NO3 T3 - PLA T4	4.886	2.81	117	1.737	0.0851
NO3 T3 - NO3 T5	-5.186	2.81	117	-1.843	0.0678
NO3 T3 - PLA T5	5.150	2.81	117	1.831	0.0697
PLA T3 - NO3 T4	-3.571	2.81	117	-1.269	0.2068
PLA T3 - PLA T4	0.757	2.81	117	0.269	0.7883
PLA T3 - NO3 T5	-9.314	2.81	117	-3.311	0.0012
PLA T3 - PLA T5	1.021	2.81	117	0.363	0.7172
NO3 T4 - PLA T4	4.329	2.81	117	1.539	0.1266
NO3 T4 - NO3 T5	-5.743	2.81	117	-2.041	0.0435
NO3 T4 - PLA T5	4.593	2.81	117	1.633	0.1052
PLA T4 - NO3 T5	-10.071	2.81	117	-3.580	0.0005
PLA T4 - PLA T5	0.264	2.81	117	0.094	0.9253
NO3 T5 - PLA T5	10.336	2.81	117	3.674	0.0004

RMSSD (Acute)

Group	Time	emmean	SE	df	lower.CL	upper.CL
NO3	T1	10.15	1.92	34.2	6.25	14.1
PLA	T1	9.07	1.92	34.2	5.17	13.0
NO3	T2	11.44	1.92	34.2	7.54	15.3
PLA	T2	9.79	1.92	34.2	5.88	13.7
NO3	T3	12.66	1.92	34.2	8.76	16.6
PLA	T3	11.26	1.92	34.2	7.35	15.2
NO3	T4	13.00	1.92	34.2	9.10	16.9
PLA	T4	10.16	1.92	34.2	6.25	14.1
NO3	T5	13.96	1.92	34.2	10.06	17.9
PLA	T5	10.39	1.92	34.2	6.48	14.3

Linear mixed model

contrast	estimate	SE	df	t.ratio	p.value
NO3 T1 - PLA T1	1.07857	1.81	117	0.597	0.5514
NO3 T1 - NO3 T2	-1.29286	1.81	117	-0.716	0.4753
NO3 T1 - PLA T2	0.36429	1.81	117	0.202	0.8404
NO3 T1 - NO3 T3	-2.51429	1.81	117	-1.393	0.1664
NO3 T1 - PLA T3	-1.10714	1.81	117	-0.613	0.5409
NO3 T1 - NO3 T4	-2.85000	1.81	117	-1.579	0.1171
NO3 T1 - PLA T4	-0.00714	1.81	117	-0.004	0.9968
NO3 T1 - NO3 T5	-3.81429	1.81	117	-2.113	0.0367
NO3 T1 - PLA T5	-0.23571	1.81	117	-0.131	0.8963
PLA T1 - NO3 T2	-2.37143	1.81	117	-1.314	0.1916
PLA T1 - PLA T2	-0.71429	1.81	117	-0.396	0.6931
PLA T1 - NO3 T3	-3.59286	1.81	117	-1.990	0.0489
PLA T1 - PLA T3	-2.18571	1.81	117	-1.211	0.2285
PLA T1 - NO3 T4	-3.92857	1.81	117	-2.176	0.0316
PLA T1 - PLA T4	-1.08571	1.81	117	-0.601	0.5487
PLA T1 - NO3 T5	-4.89286	1.81	117	-2.710	0.0077
PLA T1 - PLA T5	-1.31429	1.81	117	-0.728	0.4681
NO3 T2 - PLA T2	1.65714	1.81	117	0.918	0.3606
NO3 T2 - NO3 T3	-1.22143	1.81	117	-0.677	0.5000
NO3 T2 - PLA T3	0.18571	1.81	117	0.103	0.9182
NO3 T2 - NO3 T4	-1.55714	1.81	117	-0.863	0.3902
NO3 T2 - PLA T4	1.28571	1.81	117	0.712	0.4778
NO3 T2 - NO3 T5	-2.52143	1.81	117	-1.397	0.1652
NO3 T2 - PLA T5	1.05714	1.81	117	0.586	0.5593
PLA T2 - NO3 T3	-2.87857	1.81	117	-1.594	0.1135
PLA T2 - PLA T3	-1.47143	1.81	117	-0.815	0.4167
PLA T2 - NO3 T4	-3.21429	1.81	117	-1.780	0.0776
PLA T2 - PLA T4	-0.37143	1.81	117	-0.206	0.8374
PLA T2 - NO3 T5	-4.17857	1.81	117	-2.315	0.0224
PLA T2 - PLA T5	-0.60000	1.81	117	-0.332	0.7402
NO3 T3 - PLA T3	1.40714	1.81	117	0.779	0.4373
NO3 T3 - NO3 T4	-0.33571	1.81	117	-0.186	0.8528
NO3 T3 - PLA T4	2.50714	1.81	117	1.389	0.1676
NO3 T3 - NO3 T4	-0.33571	1.81	117	-0.186	0.8528
NO3 T3 - PLA T4	2.50714	1.81	117	1.389	0.1676
NO3 T3 - NO3 T5	-1.30000	1.81	117	-0.720	0.4729
NO3 T3 - PLA T5	2.27857	1.81	117	1.262	0.2094
PLA T3 - NO3 T4	-1.74286	1.81	117	-0.965	0.3363
PLA T3 - PLA T4	1.10000	1.81	117	0.609	0.5435
PLA T3 - NO3 T5	-2.70714	1.81	117	-1.500	0.1364
PLA T3 - PLA T5	0.87143	1.81	117	0.483	0.6302
NO3 T4 - PLA T4	2.84286	1.81	117	1.575	0.1180
NO3 T4 - NO3 T5	-0.96429	1.81	117	-0.534	0.5943
NO3 T4 - PLA T5	2.61429	1.81	117	1.448	0.1503
PLA T4 - NO3 T5	-3.80714	1.81	117	-2.109	0.0371
PLA T4 - PLA T5	-0.22857	1.81	117	-0.127	0.8995
NO3 T5 - PLA T5	3.57857	1.81	117	1.982	0.0498

HF (Acute)

Group	Time	emmean	SE	df	lower.CL	upper.CL
NO3	T1	48.4	18.6	71.2	11.242	85.5
PLA	T1	43.7	18.6	71.2	6.600	80.8
NO3	T2	65.9	18.6	71.2	28.742	103.0
PLA	T2	41.3	18.6	71.2	4.171	78.4
NO3	T3	77.4	18.6	71.2	40.314	114.5
PLA	T3	37.3	18.6	71.2	0.171	74.4
NO3	T4	55.4	18.6	71.2	18.242	92.5
PLA	T4	46.2	18.6	71.2	9.100	83.3
NO3	T5	76.8	18.6	71.2	39.671	113.9
PLA	T5	66.2	18.6	71.2	29.100	103.3

Linear mixed model

contrast	estimate	SE	df	t.ratio	p.value
NO3 T1 - PLA T1	4.643	22	117	0.211	0.8331
NO3 T1 - NO3 T2	-17.500	22	117	-0.796	0.4276
NO3 T1 - PLA T2	7.071	22	117	0.322	0.7482
NO3 T1 - NO3 T3	-29.071	22	117	-1.323	0.1885
NO3 T1 - PLA T3	11.071	22	117	0.504	0.6154
NO3 T1 - NO3 T4	-7.000	22	117	-0.318	0.7507
NO3 T1 - PLA T4	2.143	22	117	0.097	0.9225
NO3 T1 - NO3 T5	-28.429	22	117	-1.293	0.1984
NO3 T1 - PLA T5	-17.857	22	117	-0.812	0.4182
PLA T1 - NO3 T2	-22.143	22	117	-1.007	0.3158
PLA T1 - PLA T2	2.429	22	117	0.110	0.9122
PLA T1 - NO3 T3	-33.714	22	117	-1.534	0.1278
PLA T1 - PLA T3	6.429	22	117	0.292	0.7704
PLA T1 - NO3 T4	-11.643	22	117	-0.530	0.5973
PLA T1 - PLA T4	-2.500	22	117	-0.114	0.9096
PLA T1 - NO3 T5	-33.071	22	117	-1.505	0.1351
PLA T1 - PLA T5	-22.500	22	117	-1.024	0.3081
NO3 T2 - PLA T2	24.571	22	117	1.118	0.2659
NO3 T2 - NO3 T3	-11.571	22	117	-0.526	0.5996
NO3 T2 - PLA T3	28.571	22	117	1.300	0.1962
NO3 T2 - NO3 T4	10.500	22	117	0.478	0.6338
NO3 T2 - PLA T4	19.643	22	117	0.894	0.3733
NO3 T2 - NO3 T5	-10.929	22	117	-0.497	0.6200
NO3 T2 - PLA T5	-0.357	22	117	-0.016	0.9871
PLA T2 - NO3 T3	-36.143	22	117	-1.644	0.1028
PLA T2 - PLA T3	4.000	22	117	0.182	0.8559
PLA T2 - NO3 T4	-14.071	22	117	-0.640	0.5233
PLA T2 - PLA T4	-4.929	22	117	-0.224	0.8230
PLA T2 - NO3 T5	-35.500	22	117	-1.615	0.1090
PLA T2 - PLA T5	-24.929	22	117	-1.134	0.2591
NO3 T3 - PLA T3	40.143	22	117	1.826	0.0704
NO3 T3 - NO3 T4	22.071	22	117	1.004	0.3174
NO3 T3 - PLA T4	31.214	22	117	1.420	0.1582
NO3 T3 - NO3 T5	0.643	22	117	0.029	0.9767
NO3 T3 - PLA T5	11.214	22	117	0.510	0.6109
PLA T3 - NO3 T4	-18.071	22	117	-0.822	0.4127
PLA T3 - PLA T4	-8.929	22	117	-0.406	0.6853
PLA T3 - NO3 T5	-39.500	22	117	-1.797	0.0749
PLA T3 - PLA T5	-28.929	22	117	-1.316	0.1907
NO3 T4 - PLA T4	9.143	22	117	0.416	0.6782
NO3 T4 - NO3 T5	-21.429	22	117	-0.975	0.3316
NO3 T4 - PLA T5	-10.857	22	117	-0.494	0.6223
PLA T4 - NO3 T5	-30.571	22	117	-1.391	0.1669
PLA T4 - PLA T5	-20.000	22	117	-0.910	0.3647
NO3 T5 - PLA T5	10.571	22	117	0.481	0.6315

SDNN (7-day)

Group	Time	emmean	SE	df	lower.CL	upper.CL
NO3	T1	14.9	2.04	57.2	10.79	19.0
PLA	T1	12.6	1.99	53.3	8.61	16.6
NO3	T2	19.3	2.04	57.2	15.26	23.4
PLA	T2	12.6	1.99	53.3	8.58	16.5
NO3	T3	17.3	2.04	57.2	13.23	21.4
PLA	T3	13.0	1.99	53.3	9.01	17.0
NO3	T4	19.7	2.04	57.2	15.59	23.8
PLA	T4	11.0	1.99	53.3	7.00	15.0
NO3	T5	20.0	2.04	57.2	15.90	24.1
PLA	T5	11.9	1.99	53.3	7.87	15.8

Linear mixed model

contrast	estimate	SE	df	t.ratio	p.value
NO3 T1 - PLA T1	2.2781	2.24	112	1.019	0.3105
NO3 T1 - NO3 T2	-4.4692	2.27	112	-1.969	0.0514
NO3 T1 - PLA T2	2.3067	2.24	112	1.032	0.3045
NO3 T1 - NO3 T3	-2.4385	2.27	112	-1.074	0.2850
NO3 T1 - PLA T3	1.8781	2.24	112	0.840	0.4027
NO3 T1 - NO3 T4	-4.8000	2.27	112	-2.115	0.0367
NO3 T1 - PLA T4	3.8852	2.24	112	1.738	0.0850
NO3 T1 - NO3 T5	-5.1154	2.27	112	-2.253	0.0262
NO3 T1 - PLA T5	3.0138	2.24	112	1.348	0.1804
PLA T1 - NO3 T2	-6.7473	2.24	112	-3.018	0.0032
PLA T1 - PLA T2	0.0286	2.19	112	0.013	0.9896
PLA T1 - NO3 T3	-4.7166	2.24	112	-2.109	0.0371
PLA T1 - PLA T3	-0.4000	2.19	112	-0.183	0.8552
PLA T1 - NO3 T4	-7.0781	2.24	112	-3.166	0.0020
PLA T1 - PLA T4	1.6071	2.19	112	0.735	0.4640
PLA T1 - NO3 T5	-7.3935	2.24	112	-3.307	0.0013
PLA T1 - PLA T5	0.7357	2.19	112	0.336	0.7372
NO3 T2 - PLA T2	6.7759	2.24	112	3.030	0.0030
NO3 T2 - NO3 T3	2.0308	2.27	112	0.895	0.3729
NO3 T2 - PLA T3	6.3473	2.24	112	2.839	0.0054
NO3 T2 - NO3 T4	-0.3308	2.27	112	-0.146	0.8844
NO3 T2 - PLA T4	8.3545	2.24	112	3.736	0.0003
NO3 T2 - NO3 T5	-0.6462	2.27	112	-0.285	0.7764
NO3 T2 - PLA T5	7.4831	2.24	112	3.347	0.0011
PLA T2 - NO3 T3	-4.7451	2.24	112	-2.122	0.0360
PLA T2 - PLA T3	-0.4286	2.19	112	-0.196	0.8450
PLA T2 - NO3 T4	-7.1067	2.24	112	-3.178	0.0019
PLA T2 - PLA T4	1.5786	2.19	112	0.722	0.4720
PLA T2 - NO3 T5	-7.4221	2.24	112	-3.319	0.0012
PLA T2 - PLA T5	0.7071	2.19	112	0.323	0.7471
NO3 T3 - PLA T3	4.3166	2.24	112	1.931	0.0561
NO3 T3 - NO3 T4	-2.3615	2.27	112	-1.040	0.3004
NO3 T3 - PLA T4	6.3237	2.24	112	2.828	0.0055
NO3 T3 - PLA T4	6.3237	2.24	112	2.828	0.0055
NO3 T3 - NO3 T5	-2.6769	2.27	112	-1.179	0.2408
NO3 T3 - PLA T5	5.4523	2.24	112	2.438	0.0163
PLA T3 - NO3 T4	-6.6781	2.24	112	-2.987	0.0035
PLA T3 - PLA T4	2.0071	2.19	112	0.918	0.3608
PLA T3 - NO3 T5	-6.9935	2.24	112	-3.128	0.0022
PLA T3 - PLA T5	1.1357	2.19	112	0.519	0.6046
NO3 T4 - PLA T4	8.6852	2.24	112	3.884	0.0002
NO3 T4 - NO3 T5	-0.3154	2.27	112	-0.139	0.8898
NO3 T4 - PLA T5	7.8138	2.24	112	3.495	0.0007
PLA T4 - NO3 T5	-9.0006	2.24	112	-4.025	0.0001
PLA T4 - PLA T5	-0.8714	2.19	112	-0.398	0.6911
NO3 T5 - PLA T5	8.1292	2.24	112	3.636	0.0004

RMSSD (7-day)

Group	Time	emmean	SE	df	lower.CL	upper.CL
NO3	T1	10.71	1.71	43.0	7.27	14.2
PLA	T1	8.64	1.67	40.1	5.26	12.0
NO3	T2	16.03	1.71	43.0	12.58	19.5
PLA	T2	10.32	1.67	40.1	6.94	13.7
NO3	T3	15.14	1.71	43.0	11.69	18.6
PLA	T3	9.94	1.67	40.1	6.56	13.3
NO3	T4	16.73	1.71	43.0	13.28	20.2
PLA	T4	9.24	1.67	40.1	5.86	12.6
NO3	T5	14.99	1.71	43.0	11.54	18.4
PLA	T5	10.17	1.67	40.1	6.79	13.5

Linear mixed model

contrast	estimate	SE	df	t.ratio	p.value
NO3 T1 - PLA T1	2.078	1.72	112	1.209	0.2292
NO3 T1 - NO3 T2	-5.315	1.74	112	-3.047	0.0029
NO3 T1 - PLA T2	0.392	1.72	112	0.228	0.8199
NO3 T1 - NO3 T3	-4.423	1.74	112	-2.536	0.0126
NO3 T1 - PLA T3	0.778	1.72	112	0.453	0.6517
NO3 T1 - NO3 T4	-6.015	1.74	112	-3.448	0.0008
NO3 T1 - PLA T4	1.478	1.72	112	0.860	0.3916
NO3 T1 - NO3 T5	-4.277	1.74	112	-2.452	0.0158
NO3 T1 - PLA T5	0.542	1.72	112	0.315	0.7530
PLA T1 - NO3 T2	-7.393	1.72	112	-4.302	<.0001
PLA T1 - PLA T2	-1.686	1.68	112	-1.003	0.3181
PLA T1 - NO3 T3	-6.501	1.72	112	-3.783	0.0003
PLA T1 - PLA T3	-1.300	1.68	112	-0.773	0.4409
PLA T1 - NO3 T4	-8.093	1.72	112	-4.709	<.0001
PLA T1 - PLA T4	-0.600	1.68	112	-0.357	0.7218
PLA T1 - NO3 T5	-6.355	1.72	112	-3.698	0.0003
PLA T1 - PLA T5	-1.536	1.68	112	-0.914	0.3629
NO3 T2 - PLA T2	5.708	1.72	112	3.321	0.0012
NO3 T2 - NO3 T3	0.892	1.74	112	0.512	0.6100
NO3 T2 - PLA T3	6.093	1.72	112	3.546	0.0006
NO3 T2 - NO3 T4	-0.700	1.74	112	-0.401	0.6890
NO3 T2 - PLA T4	6.793	1.72	112	3.953	0.0001
NO3 T2 - NO3 T5	1.038	1.74	112	0.595	0.5528
NO3 T2 - PLA T5	5.858	1.72	112	3.408	0.0009
PLA T2 - NO3 T3	-4.815	1.72	112	-2.802	0.0060
PLA T2 - PLA T3	0.386	1.68	112	0.229	0.8189
PLA T2 - NO3 T4	-6.408	1.72	112	-3.728	0.0003
PLA T2 - PLA T4	1.086	1.68	112	0.646	0.5197
PLA T2 - NO3 T5	-4.669	1.72	112	-2.717	0.0076
PLA T2 - PLA T5	0.150	1.68	112	0.089	0.9291
NO3 T3 - PLA T3	5.201	1.72	112	3.026	0.0031
NO3 T3 - NO3 T4	-1.592	1.74	112	-0.913	0.3633
NO3 T3 - PLA T4	5.901	1.72	112	3.434	0.0008
NO3 T3 - NO3 T5	0.146	1.74	112	0.084	0.9334
NO3 T3 - PLA T5	4.965	1.72	112	2.889	0.0046
PLA T3 - NO3 T4	-6.793	1.72	112	-3.953	0.0001
PLA T3 - PLA T4	0.700	1.68	112	0.416	0.6779
PLA T3 - NO3 T5	-5.055	1.72	112	-2.941	0.0040
PLA T3 - PLA T5	-0.236	1.68	112	-0.140	0.8887
NO3 T4 - PLA T4	7.493	1.72	112	4.360	<.0001
NO3 T4 - NO3 T5	1.738	1.74	112	0.997	0.3211
NO3 T4 - PLA T5	6.558	1.72	112	3.816	0.0002
PLA T4 - NO3 T5	-5.755	1.72	112	-3.349	0.0011
PLA T4 - PLA T5	-0.936	1.68	112	-0.557	0.5789
NO3 T5 - PLA T5	4.819	1.72	112	2.804	0.0059

HF (7-day)

Group	Time	emmean	SE	df	lower.CL	upper.CL
NO3	T1	61.5	30.3	75.9	1.06	121.8
PLA	T1	35.7	29.4	71.5	-22.93	94.4
NO3	T2	125.5	30.3	75.9	65.14	185.9
PLA	T2	82.2	29.4	71.5	23.57	140.9
NO3	T3	116.5	30.3	75.9	56.06	176.8
PLA	T3	48.1	29.4	71.5	-10.50	106.8
NO3	T4	150.5	30.3	75.9	90.14	210.9
PLA	T4	56.7	29.4	71.5	-1.93	115.4
NO3	T5	110.1	30.3	75.9	49.76	170.5
PLA	T5	44.0	29.4	71.5	-14.64	102.6

Linear mixed model

contrast	estimate	SE	df	t.ratio	p.value
NO3 T1 - PLA T1	25.74	35.7	112	0.720	0.4729
NO3 T1 - NO3 T2	-64.08	36.3	112	-1.766	0.0802
NO3 T1 - PLA T2	-20.76	35.7	112	-0.581	0.5624
NO3 T1 - NO3 T3	-55.00	36.3	112	-1.515	0.1325
NO3 T1 - PLA T3	13.31	35.7	112	0.372	0.7103
NO3 T1 - NO3 T4	-89.08	36.3	112	-2.454	0.0157
NO3 T1 - PLA T4	4.74	35.7	112	0.133	0.8948
NO3 T1 - NO3 T5	-48.69	36.3	112	-1.342	0.1824
NO3 T1 - PLA T5	17.45	35.7	112	0.488	0.6263
PLA T1 - NO3 T2	-89.81	35.7	112	-2.513	0.0134
PLA T1 - PLA T2	-46.50	35.0	112	-1.330	0.1863
PLA T1 - NO3 T3	-80.74	35.7	112	-2.259	0.0258
PLA T1 - PLA T3	-12.43	35.0	112	-0.355	0.7230
PLA T1 - NO3 T4	-114.81	35.7	112	-3.213	0.0017
PLA T1 - PLA T4	-21.00	35.0	112	-0.600	0.5494
PLA T1 - NO3 T5	-74.43	35.7	112	-2.083	0.0396
PLA T1 - PLA T5	-8.29	35.0	112	-0.237	0.8132
NO3 T2 - PLA T2	43.31	35.7	112	1.212	0.2281
NO3 T2 - NO3 T3	9.08	36.3	112	0.250	0.8030
NO3 T2 - PLA T3	77.39	35.7	112	2.165	0.0325
NO3 T2 - NO3 T4	-25.00	36.3	112	-0.689	0.4923
NO3 T2 - PLA T4	68.81	35.7	112	1.926	0.0567
NO3 T2 - NO3 T5	15.38	36.3	112	0.424	0.6724
NO3 T2 - PLA T5	81.53	35.7	112	2.281	0.0244
PLA T2 - NO3 T3	-34.24	35.7	112	-0.958	0.3401
PLA T2 - PLA T3	34.07	35.0	112	0.974	0.3320
PLA T2 - NO3 T4	-68.31	35.7	112	-1.912	0.0585
PLA T2 - PLA T4	25.50	35.0	112	0.729	0.4674
PLA T2 - NO3 T5	-27.93	35.7	112	-0.782	0.4361
PLA T2 - PLA T5	38.21	35.0	112	1.093	0.2769
NO3 T3 - PLA T3	68.31	35.7	112	1.911	0.0585
NO3 T3 - NO3 T4	-34.08	36.3	112	-0.939	0.3498
NO3 T3 - PLA T4	59.74	35.7	112	1.672	0.0974
NO3 T3 - NO3 T5	6.31	36.3	112	0.174	0.8623
NO3 T3 - PLA T5	72.45	35.7	112	2.027	0.0450
PLA T3 - NO3 T4	-102.39	35.7	112	-2.865	0.0050
PLA T3 - PLA T4	-8.57	35.0	112	-0.245	0.8068
PLA T3 - NO3 T5	-62.00	35.7	112	-1.735	0.0855
PLA T3 - PLA T5	4.14	35.0	112	0.118	0.9059
NO3 T4 - PLA T4	93.81	35.7	112	2.625	0.0099
NO3 T4 - NO3 T5	40.38	36.3	112	1.113	0.2682
NO3 T4 - PLA T5	106.53	35.7	112	2.981	0.0035
PLA T4 - NO3 T5	-53.43	35.7	112	-1.495	0.1377
